# Microglial TSPAN4‐Dependent Migrasomes Promote Pathological Retinal Neovascularization via Immune‐Vascular Crosstalk

**DOI:** 10.1002/advs.77711

**Published:** 2026-09-13

**Authors:** Jingyi Xu, Jingfan Wang, Pengfei Ge, Qinyuan Gu, Hongying Li, Yuanyuan Fan, Haiyue Xie, Yangyang Lu, Yifan Lin, Xinjing Wu, Chengkun Wang, Ping Xie, Zizhong Hu

**Affiliations:** ^1^ Department of Ophthalmology The First Affiliated Hospital of Nanjing Medical University Nanjing China; ^2^ Department of Ophthalmology Children's Hospital of Nanjing Medical University Nanjing China; ^3^ Department of Physiology School of Basic Medical Sciences Nanjing Medical University Nanjing China

**Keywords:** diabetic retinopathy, microglia, migrasomes, retinal neovascularization, TSPAN4

## Abstract

Diabetic retinopathy (DR) is a leading cause of vision loss among working‐age adults worldwide, yet the microglial mechanisms driving pathological retinal neovascularization (RNV) remain incompletely understood. By integrating single‐cell transcriptomic profiling of human fibrovascular membranes with murine disease models, we identified a TSPAN4‐associated microglial state characterized by a pro‐angiogenic and activated transcriptional program. Spatially, TSPAN4‐expressing microglia accumulated around pathological vascular tufts, and suppression of TSPAN4 attenuated RNV in the oxygen‐induced retinopathy (OIR) model. Under high‐glucose and hypoxic conditions, microglia upregulated TSPAN4 expression and triggered the biogenesis of migrasomes along retraction fibers. Microglia‐derived migrasomes were sufficient to promote endothelial angiogenic responses in vitro and pathological neovascularization in vivo. Mechanistically, transcriptomic analyses revealed that microglial migrasomes exerted pro‐angiogenic effects by activating the HIF‐1α/VEGF pathway in endothelial cells. In parallel, migrasomes reinforced a pro‐inflammatory microglial phenotype, establishing a pathogenic feed‐forward loop that amplified retinal vascular injury. Collectively, our findings define TSPAN4‐dependent migrasome formation as a critical mechanism through which microglia promote pathological RNV and uncover migrasome‐mediated communication as a previously unrecognized mode of immune‐vascular crosstalk. Targeting the microglial TSPAN4‐migrasome axis represents a promising therapeutic strategy for neovascular retinal diseases.

## Graphical abstract

Combined high‐glucose and hypoxic stress switches microglia into a TSPAN4‐dependent migrasome‐producing state. These migrasomes deliver pro‐angiogenic signals to endothelial cells, activating HIF‐1α/VEGF signaling, disrupting vascular junctions, and fueling pathological neovascularization.  This work uncovers migrasome‐mediated immune‐vascular crosstalk and exposes the microglial TSPAN4‐migrasome axis as a therapeutic vulnerability in neovascular retinal disease.

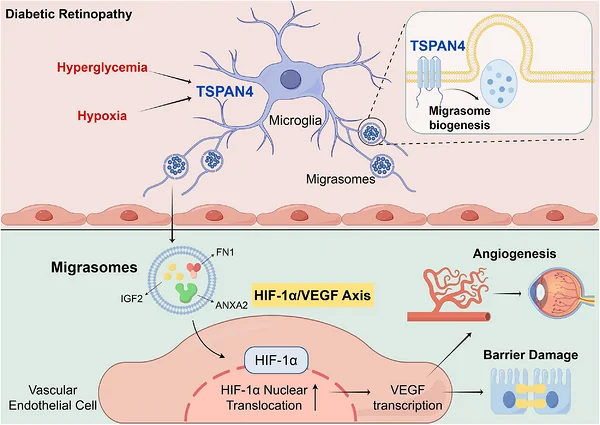

## 1 Introduction

Diabetic retinopathy (DR) is a major microvascular complication of diabetes and a leading cause of vision impairment among working‐age adults worldwide [1, 2]. Early‐stage DR is characterized by microvascular injury and disruption of the blood–retina barrier. Without timely intervention, the disease may progress to proliferative diabetic retinopathy (PDR), in which pathological retinal neovascularization (RNV) can cause vitreous hemorrhage and tractional retinal detachment [3]. Although anti–vascular endothelial growth factor (VEGF) therapy and vitrectomy have improved clinical outcomes [4, 5, 6], some patients respond inadequately, and the need for repeated intravitreal injections imposes a substantial treatment burden [7, 8, 9]. Thus, identifying novel molecular drivers of pathological angiogenesis is imperative.

Emerging evidence indicates that DR involves dysfunction of the retinal neurovascular–immune unit rather than the vasculature alone [10, 11, 12, 13]. Microglia, the resident immune cells of the retina, are pivotal orchestrators of inflammatory responses and vascular homeostasis [14, 15, 16, 17, 18]. Under physiological conditions, microglia support retinal integrity [19, 20, 21]. However, metabolic stress and hypoxia trigger a dramatic phenotypic shift [22, 23, 24]. Activated microglia migrate toward pathological sites, releasing inflammatory and pro‐angiogenic mediators that fuel vascular leakage and sprouting [25, 26, 27]. Our preliminary studies confirmed that microglia constitute a major cellular component of fibrovascular membranes (FVMs) from patients with PDR [28, 29]. While microglial activation is recognized as a critical bridge between metabolic dysregulation and vascular injury [15, 30], the precise spatiotemporal mechanisms by which activated microglia deliver concentrated pro‐angiogenic signals to the vascular endothelium beyond simple diffusion of secreted factors remain a biological enigma.

Through single‐cell RNA sequencing (scRNA‐seq) analysis of human FVMs and murine oxygen‐induced retinopathy (OIR) tissues, we identified a TSPAN4‐associated transcriptional state within the microglial compartment. Rather than defining a discrete cluster, this state was represented by a broader gene signature that partially overlapped with established activated and disease‐associated microglial programs. Among the molecular features associated with this state, TSPAN4 was of particular interest because of its established role in migrasome biogenesis. Migrasomes are membrane‐bound structures formed along retraction fibers that transport bioactive cargo and mediate spatially restricted intercellular communication [31, 32, 33]. Although migrasomes derived from other cell types have been implicated in vascular growth [34, 35], whether TSPAN4‐dependent migrasomes mediate communication between microglia and the retinal vasculature remains unexplored.

In this study, we investigated whether high‐glucose and hypoxic stress promotes TSPAN4‐dependent migrasome biogenesis in microglia and whether these migrasomes mediate pathological immune–vascular communication. Utilizing *Tspan4* ^−/−^ mice, the OIR model, and complementary in vitro microglia–endothelial systems, we found that microglial migrasomes act as potent signaling vehicles that promote endothelial angiogenic responses through activation of the HIF‐1α/VEGF signaling axis. Simultaneously, these migrasomes reinforce a pro‐inflammatory microglial phenotype, establishing a pathogenic feed‐forward loop that amplifies retinal vascular injury. Collectively, these findings identify TSPAN4‐dependent migrasome formation as a novel mode of microglia–endothelial crosstalk and position the microglial TSPAN4–migrasome axis as a promising therapeutic target for pathological retinal neovascularization.

## 2 Results

### 2.1 TSPAN4‐Associated Microglial State in FVMs and OIR Retinas

**FIGURE 1:**
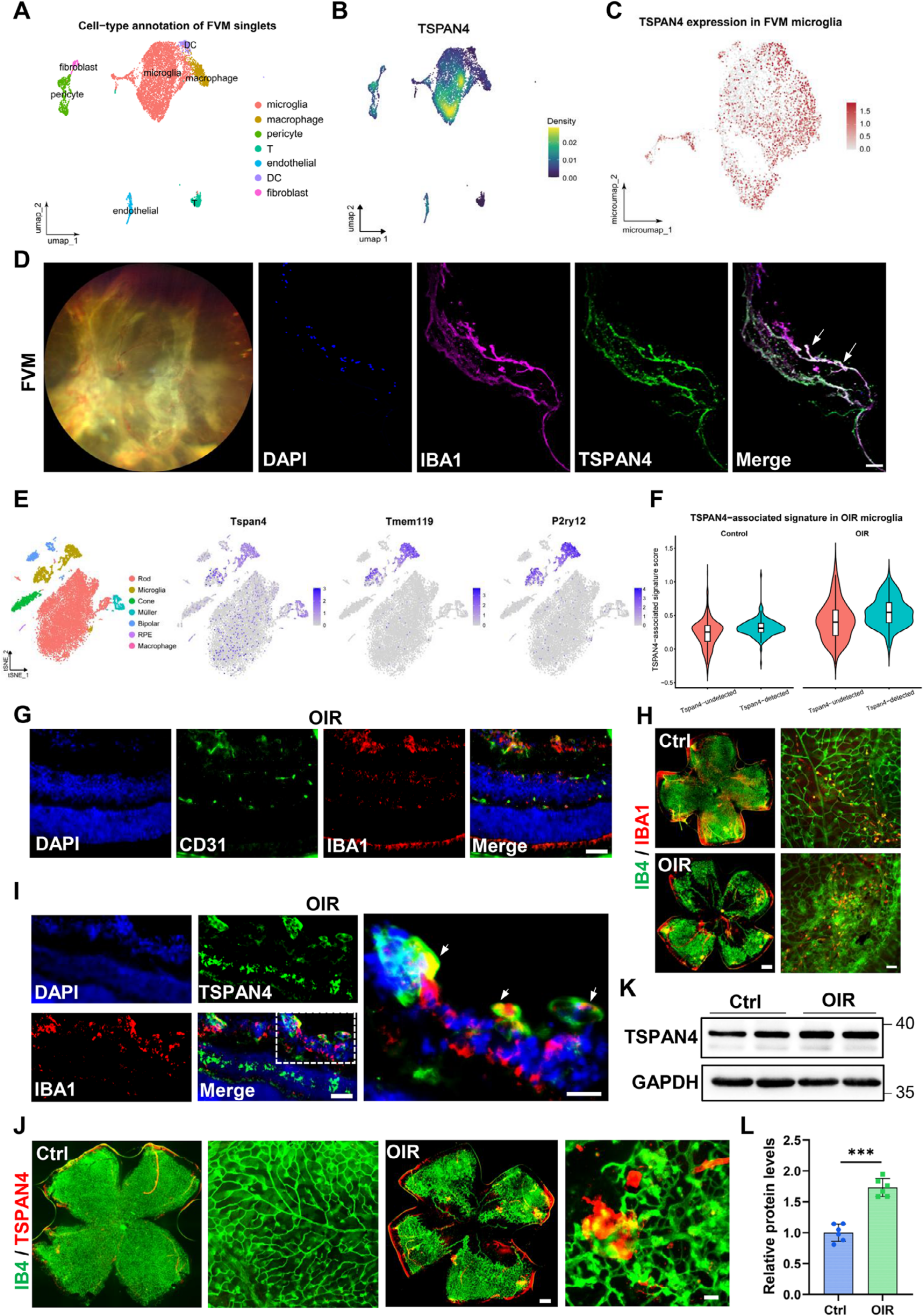
A TSPAN4‐associated microglial state in human PDR FVMs and murine OIR retinas. (A) UMAP of scRNA‐seq data from FVMs of patients with PDR, identifying major retinal cell populations. (B) Density plot of TSPAN4 expression showing the distribution of TSPAN4 expression across major FVM cell populations, with prominent expression in the microglial compartment. (C) UMAP feature plot showing the continuous distribution of TSPAN4 expression within the annotated microglial compartment. (D) Representative fundus photograph from a patient with PDR and immunofluorescence staining of TSPAN4 (green) and IBA1 (purple) in FVM sections, showing IBA1^+^TSPAN4^+^ microglia within the fibrovascular lesion. Scale bar = 100 µm. (E) Feature plots showing the expression of Tspan4 and microglial markers (P2ry12 and Tmem119) in the OIR dataset. (F) Violin plots comparing TSPAN4‐associated signature scores between Tspan4‐detected and Tspan4‐undetected microglia in control and OIR retinas. (G) Immunofluorescence staining of CD31 (green) and IBA1 (red) in OIR retinal sections, demonstrating the accumulation of microglia within and around pathological neovascular tufts. Scale bar = 50 µm. (H) Whole‐mount retinal staining with IB4 (green) and IBA1 (red); Scale bar = 300 µm; enlarged view scale bar = 50 µm. (I) Immunofluorescence staining of TSPAN4 (green) and IBA1 (red) in OIR retinal sections. Scale bar = 50 µm; enlarged view scale bar = 25 µm. (J) Whole‐mount retinal staining with IB4 (green) and TSPAN4 (red), showing increased TSPAN4 immunoreactivity within pathological neovascular tufts. Scale bar = 300 µm; enlarged view scale bar = 50 µm. (K) Western blot analysis shows upregulation of TSPAN4 protein in OIR retinas compared with controls. (L) Quantification of TSPAN4 protein levels in (K) (n = 6). Data are presented as mean ± SD. Statistical significance was determined using unpaired two‐tailed Student's t‐test. ^*^ *p* < 0.05; ^**^ *p* < 0.01; ^***^ *p* < 0.001; ^****^ *p* < 0.0001.

To characterize microglial involvement in DR, we analyzed scRNA‐seq data from FVMs of patients with PDR (Figure 1A). TSPAN4 expression was readily detected in the microglial compartment, which constituted the predominant immune population across the five FVM samples (Figure 1B; Figure S1A). Within this compartment, TSPAN4‐detected cells were distributed continuously across the UMAP and were present in all five samples (Figure 1C; Figure S1B). TSPAN4‐detected microglia consistently exhibited higher scores for a 30‑gene TSPAN4‑associated signature established from the differential expression program (Data S1; Table S1; Figure S1C, D). Comparison with established microglial programs revealed partial overlap with activated and disease‐associated signatures but no correspondence to a single canonical subtype (Figure S1E; Table S2). Immunofluorescence further identified IBA1^+^TSPAN4^+^ microglia within human fibrovascular lesions (Figure 1D), spatially linking microglial TSPAN4 expression to pathological neovascular tissue. To test whether this transcriptional association was conserved in vivo, we examined the murine OIR dataset. Tspan4 was detected in Tmem119^+^P2ry12^+^ microglia (Figure 1E), and Tspan4‐detected microglia scored higher for the TSPAN4‐associated signature than Tspan4‐undetected microglia (Figure 1F). In OIR retinas, IBA1^+^ microglia accumulated prominently within pathological neovascular tufts (Figure 1G,H), where TSPAN4 expression was readily detected (Figure 1I). Retinal flat mounts revealed pronounced enrichment of TSPAN4 within neovascular tufts compared with control retinas (Figure 1J). This upregulation was corroborated by western blot analysis, which showed significantly increased TSPAN4 protein expression in OIR retinas (Figure 1K,L). Importantly, these tissue‐level validations independently confirmed the scRNA‐seq findings, excluding the possibility that the observed TSPAN4 signals arose merely from dissociation artifacts. Together, these findings establish that TSPAN4‐expressing microglia are closely associated with pathological neovascular lesions, a spatial association that prompted us to further investigate their functional contribution in retinal neovascularization.

### 2.2 Tspan4 Deficiency Attenuates Pathological Angiogenesis

**FIGURE 2:**
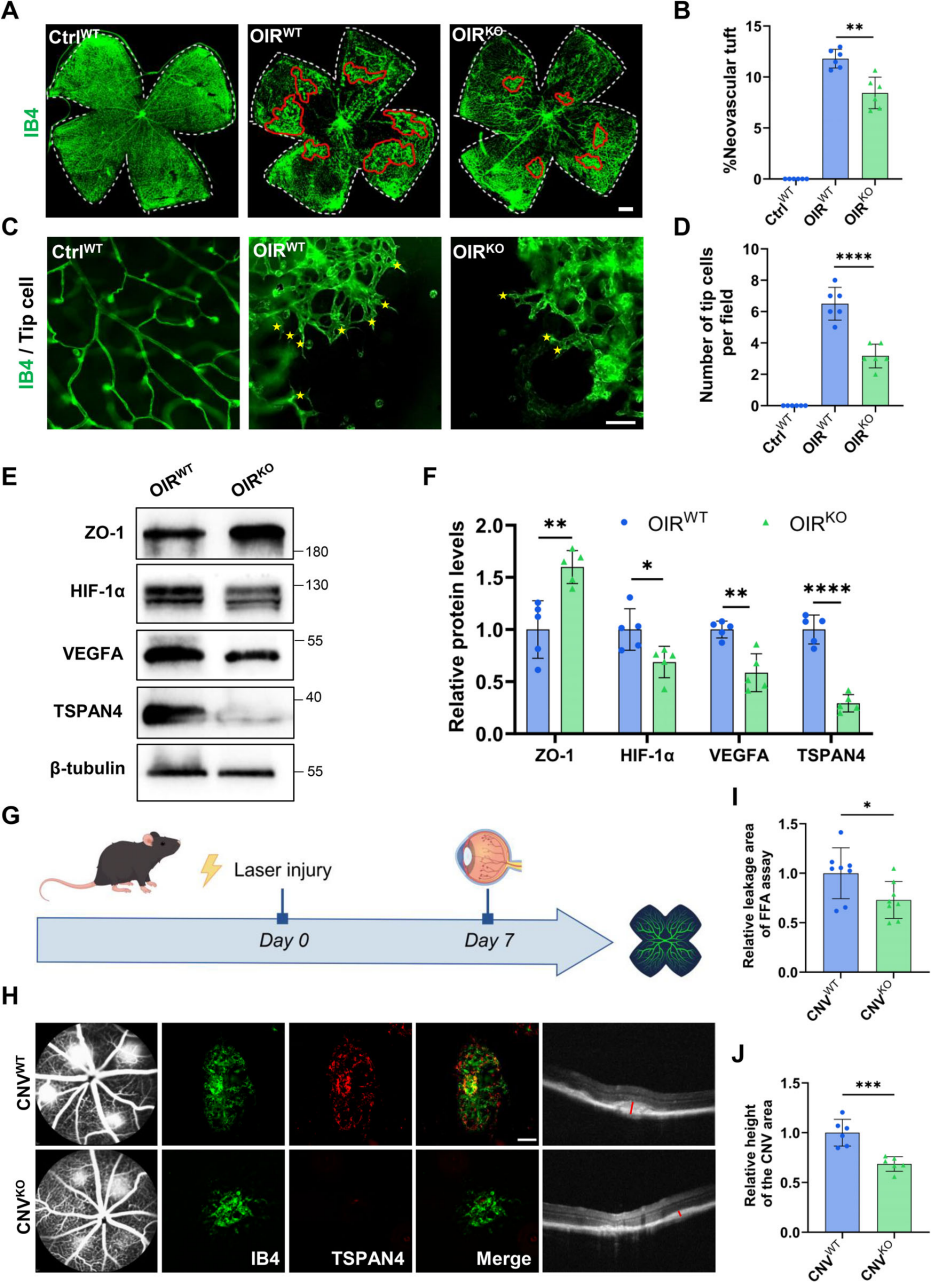
TSPAN4 deficiency suppresses pathological neovascularization. (A) Retinal flat‐mount staining with IB4 in Ctrl^WT^, OIR^WT^, and OIR^KO^ mice, showing reduced pathological neovascularization in OIR^KO^ retinas. Scale bar = 300 µm. (B) Quantification of neovascular area in retinas shown in (A) (n = 6). (C) Representative images of endothelial tip cells (yellow asterisks) in Ctrl^WT^, OIR^WT^, and OIR^KO^ retinas. Scale bar = 50 µm. (D) Quantification of tip cell number in (C) (n = 6). (E) Western blot analysis of retinal proteins in OIR^KO^ and OIR^WT^ mice, indicating reduced pro‐angiogenic signaling, enhanced endothelial barrier integrity, and successful knockout. (F) Quantification of protein expression levels shown in (E) (n = 5). (G) Schematic illustration of the laser‐induced CNV model. (H) Representative FFA imaging (left), choroidal flat‐mounts (IB4 and TSPAN4 staining, middle), and OCT images (right) of CNV lesions in WT and KO mice. Scale bar = 100 µm. (I) Quantification of fluorescein leakage area in CNV lesions shown in (H) (n = 8). (J) Quantification of CNV lesion height from OCT images in (H) (n = 6). Data are presented as mean ± SD. Statistical significance was determined using unpaired two‐tailed Student's *t*‐test. ^*^ *p* < 0.05; ^**^ *p* < 0.01; ^***^ *p* < 0.001; ^****^ *p* < 0.0001.

To determine the functional contribution of TSPAN4 to pathological angiogenesis, we compared RNV in wild‐type (WT) and global Tspan4 knockout (KO) mice using the OIR model. Global Tspan4 deletion was validated as shown in Figure S2. IB4 staining of retinal flat mounts revealed extensive pathological neovascularization in OIR^WT^ retinas, whereas neovascular tuft formation was markedly reduced in OIR^KO^ retinas (Figure 2A,B). The number of endothelial tip cells, an indicator of active angiogenic sprouting, was also significantly lower in OIR^KO^ retinas (Figure 2C,D). We next examined angiogenesis‐ and barrier‐associated protein expression. Western blot analysis revealed increased ZO‐1 expression and reduced HIF‐1α and VEGFA expression in OIR^KO^ retinas. TSPAN4 protein was undetectable in the knockout group, confirming the efficacy of Tspan4 deletion (Figure 2E,F). We further evaluated the effect of Tspan4 deficiency in a laser‐induced choroidal neovascularization (CNV) model (Figure 2G). Fundus fluorescein angiography revealed reduced vascular leakage in CNV^KO^ mice, while IB4 staining of choroidal flat mounts showed smaller CNV lesions than those in WT mice (Figure 2H). Optical coherence tomography further confirmed a significant reduction in CNV lesion height in CNV^KO^ mice (Figure 2I,J). These results demonstrate that global Tspan4 deficiency attenuates pathological neovascularization across both OIR and CNV models.

### 2.3 Microglia Enhance Endothelial Angiogenic Responses in a TSPAN4‐Dependent Manner

**FIGURE 3:**
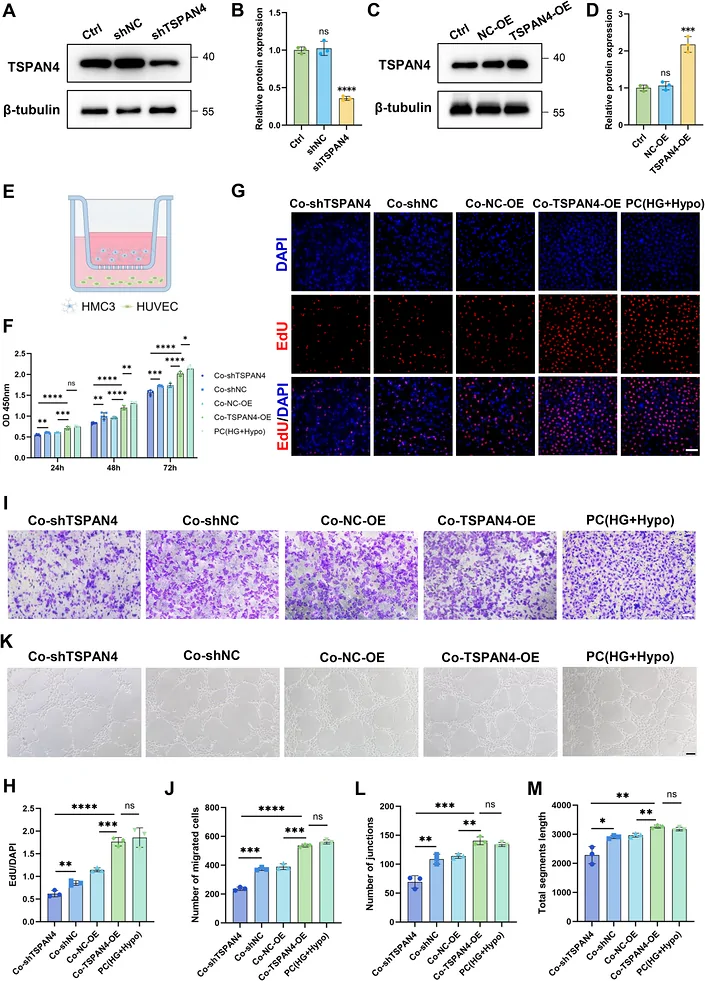
Microglia Enhance Endothelial Angiogenic Responses in a TSPAN4‐dependent Manner. (A) Western blot analysis of TSPAN4 expression in HMC3 cells transduced with shNC (non‑targeting control) or shTSPAN4 lentiviruses. (B) Quantification of TSPAN4 protein expression in (A). (C) Western blot analysis of TSPAN4 expression in HMC3 cells transduced with control vector (NC‐OE) or TSPAN4‑overexpression (TSPAN4‐OE) lentiviruses. (D) Quantification of TSPAN4 protein levels in (C). (E) Schematic illustration of the Transwell co‐culture system, in which HMC3 cells with the indicated TSPAN4 manipulations were seeded in the upper chamber and HUVECs in the lower chamber. (F) CCK‐8 assay showing HUVEC cellular activity at 24, 48, and 72 h after co‐culture with the indicated HMC3 groups. HUVECs directly exposed to HG and hypoxia were included as a positive control (PC) benchmark. (G) Representative EdU staining of HUVECs following co‐culture with shNC, shTSPAN4, NC‑OE, or TSPAN4‑OE microglia. Scale bar = 100 µm. (H) Quantification of EdU‐positive HUVECs in (G). (I) Representative Transwell migration assay of HUVECs following co‐culture with the indicated HMC3 groups. Scale bar = 100 µm. (J) Quantification of migrated HUVECs in (I). (K) Representative tube formation assay of HUVECs after co‐culture with TSPAN4‐manipulated microglia. Scale bar = 200 µm. (L) Quantification of the number of junctions in (K). (M) Quantification of total segment length in (K). Data are presented as mean ± SD. Comparisons among multiple groups were performed using one‐way or two‐way ANOVA followed by Tukey's post–hoc test, as appropriate. ^*^ *p* < 0.05; ^**^ *p* < 0.01; ^***^ *p* < 0.001; ^****^ *p* < 0.0001.

To investigate whether microglial TSPAN4 modulates endothelial angiogenic responses, Human Microglia Clone 3 (HMC3) cells were subjected to lentiviral‐mediated TSPAN4 knockdown or overexpression, and the efficiency of TSPAN4 manipulation was confirmed as shown in Figure 3A–D. Human Umbilical Vein Endothelial Cells (HUVECs) were subsequently co‐cultured with the differently transduced HMC3 cells using a Transwell system (Figure 3E). HUVECs directly exposed to HG and hypoxia were included as a positive control (PC) for endothelial angiogenic activation. Endothelial proliferation and cellular activity were assessed using EdU incorporation and CCK‐8 assays, respectively. Compared with the corresponding vector controls, co‐culture with TSPAN4‐knockdown HMC3 cells reduced HUVEC proliferation and activity, whereas TSPAN4 overexpression enhanced both responses to levels comparable to those of the positive control (Figure 3F–H). Transwell migration assays similarly showed reduced endothelial migration following co‐culture with TSPAN4‐knockdown microglia and increased migration in response to TSPAN4‐overexpressing microglia (Figure 3I,J). In tube formation assays, HUVECs co‐cultured with TSPAN4‐overexpressing microglia formed more extensive vascular networks, with increased junction numbers and total segment length, whereas TSPAN4 knockdown produced the opposite effect (Figure 3K–M). Together, these findings demonstrate that TSPAN4 expression in microglia modulates endothelial proliferative, migratory, and angiogenic responses.

### 2.4 Hyperglycemic and Hypoxic Stress Induce TSPAN4 Upregulation and Migrasome Biogenesis

**FIGURE 4:**
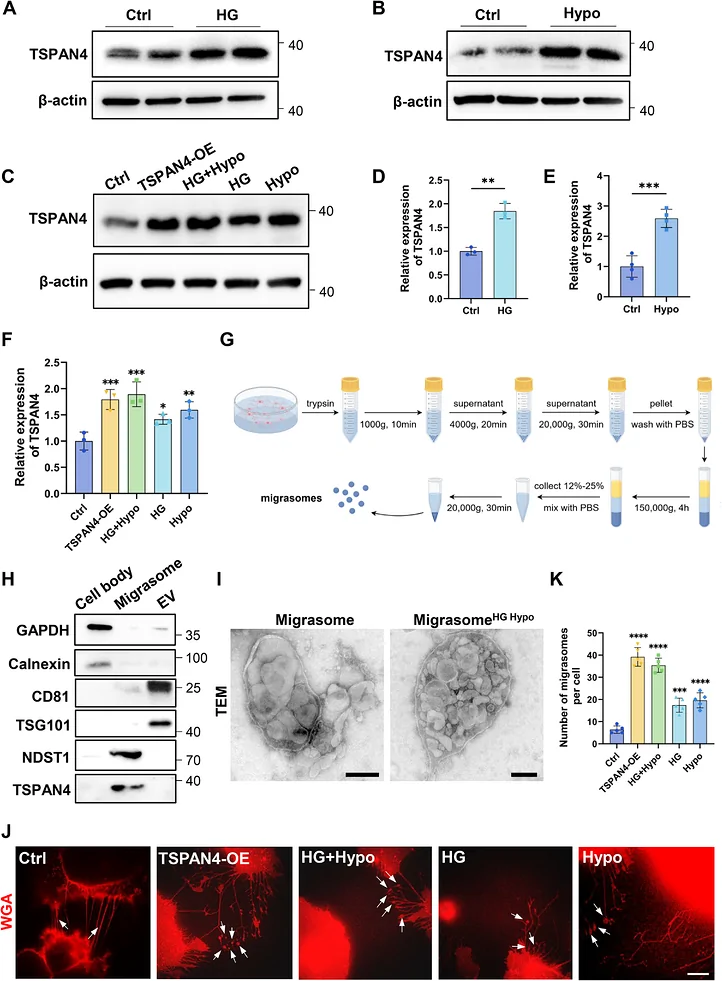
High‐glucose and hypoxic stress increase TSPAN4 expression and migrasome formation in microglia. (A, B) Western blot analysis of TSPAN4 expression in HMC3 cells cultured under HG or hypoxic conditions. (C) Western blot analysis of TSPAN4 expression in HMC3 cells under control, TSPAN4‐OE, HG + Hypo, HG and Hypo conditions. (D‐F) Quantification of TSPAN4 protein levels in (A–C), respectively. (G) Schematic illustration of migrasome isolation. (H) Western blot analysis of marker proteins in cell bodies (GAPDH^+^, Calnexin^+^), migrasomes (TSPAN4^+^, NDST1^+^), and EVs (CD81^+^, TSG101^+^). (I) TEM images of isolated migrasomes showing characteristic large vesicular structures containing multiple intraluminal vesicles. Scale bar = 200 nm. (J) Representative live‐cell images of WGA‐labeled HMC3 cells showing migrasomes formed along retraction fibers. TSPAN4‐OE was included as a positive control for migrasome formation. Scale bar = 10 µm. (K) Quantification of migrasomes per cell. Data are presented as mean ± SD. Two‐group comparisons were analyzed by unpaired two‐tailed Student's *t*‐test; multi‐group comparisons were analyzed by one‐way or two‐way ANOVA followed by Tukey's post–hoc test, as appropriate. ^*^ *p* < 0.05; ^**^ *p* < 0.01; ^***^ *p* < 0.001; ^****^ *p* < 0.0001.

Given the established role of TSPAN4 in migrasome biogenesis, we examined whether DR‐related metabolic and hypoxic stress regulates microglial TSPAN4 expression and migrasome formation. HMC3 cells were exposed to HG or hypoxic (Hypo) conditions, either alone or in combination. HG and hypoxia individually increased TSPAN4 protein expression (Figure 4A,B,D and E), whereas combined HG and hypoxic stress induced a further increase compared with either stimulus alone (Figure 4C,F). Using TSPAN4‐OE as a positive reference, the TSPAN4 level induced by HG + Hypo was comparable to that in the TSPAN4‐OE group. Migrasomes were subsequently isolated using sequential differential and density‐gradient centrifugation (Figure 4G). Western blot analysis showed enrichment of the migrasome markers TSPAN4 and NDST1 in the migrasome fraction, with minimal detection of the cell‐associated markers GAPDH and calnexin and the EV‐associated markers CD81 and TSG101 (Figure 4H). Transmission electron microscopy (TEM) revealed typical pomegranate‐like vesicular structures [36, 37, 38], which were more abundant under HG and hypoxic conditions (Figure 4I). Live‐cell imaging of WGA‐stained HMC3 cells further demonstrated increased migrasome formation along retraction fibers under combined HG and hypoxic stress. The magnitude of this response approached that induced by the TSPAN4‐OE positive control, indicating a robust effect of combined HG and hypoxic stress on migrasome formation (Figure 4J,K). Proteomic profiling of migrasomes derived from HG‐ and hypoxia‐treated microglia revealed a cargo profile enriched in angiogenesis‐ and matrix‐remodeling‐associated proteins, including FN1, THBS1, ECM1, IGF2, and ANXA2/5 (Figure S3). Collectively, these findings indicate that combined HG and hypoxic stress upregulates TSPAN4, enhances TSPAN4‐dependent migrasome formation, and reshapes the migrasomal cargo toward a pro‐angiogenic profile.

### 2.5 Microglia‐Derived Migrasomes Promote Pathological Retinal Neovascularization

**FIGURE 5:**
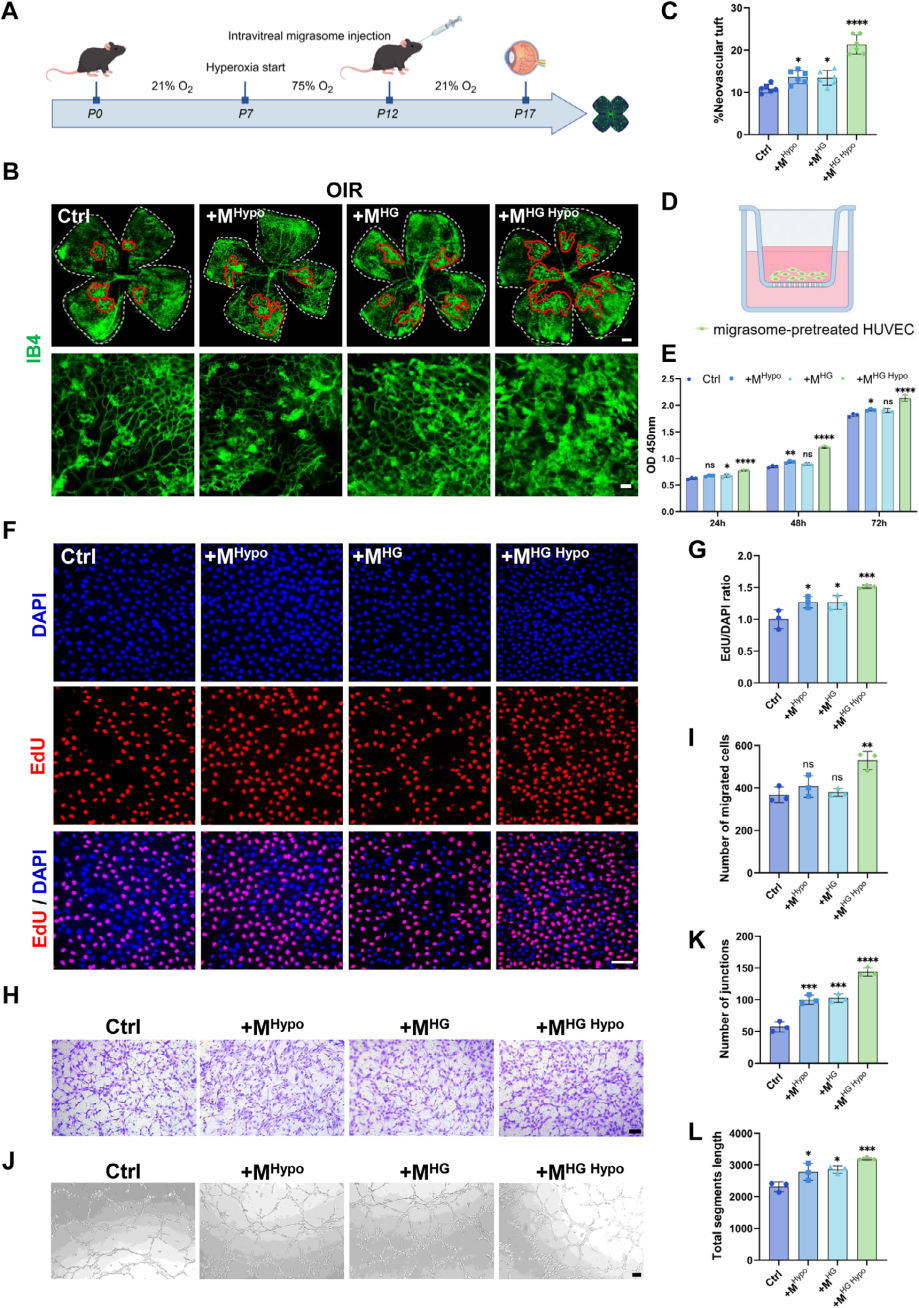
Microglia‐derived migrasomes are sufficient to enhance pathological RNV. (A) Schematic of the OIR experiment showing intravitreal administration of BV2‐derived migrasomes at P12. (B) Representative IB4‐stained retinal flat mounts showing pathological neovascular tufts following administration of migrasomes derived from control, HG‐treated, hypoxia‐treated, or combined HG + Hypo‐treated BV2 cells. Scale bar = 300 µm. Enlarged views of pathological neovascular tufts are shown below. Scale bar = 50 µm. (C) Quantification of neovascular tuft area in (B) (n = 6). (D) Schematic illustration of the Transwell migration assay using HUVECs pretreated with HMC3‐derived migrasomes. (E) CCK‐8 assay shows that only combined‐stress migrasomes significantly increase endothelial cellular activity at 24, 48, and 72 h. (F) Representative EdU staining of HUVECs treated with migrasomes. Scale bar = 100 µm. (G) Quantification of EdU‐positive cells in (F). (H) Transwell migration assay indicates that HUVEC motility is significantly enhanced by combined‐stress migrasomes. Scale bar = 200 µm. (I) Quantification of migrated cells in (H). (J) Tube formation assay shows that all migrasome‐treated groups promote network formation, with the strongest effect observed in the combined high‐glucose plus hypoxia group. Scale bar = 200 µm. (K) Quantification of junction numbers in (J). (L) Quantification of total segment length in (J). Data are presented as mean ± SD. Comparisons among multiple groups were performed using one‐way or two‐way ANOVA followed by Tukey's post–hoc test, as appropriate. ^*^ *p* < 0.05; ^**^ *p* < 0.01; ^***^ *p* < 0.001; ^****^ *p* < 0.0001.

To verify whether microglia‐derived migrasomes are taken up by endothelial cells, PKH26‐labeled migrasomes isolated from stressed microglia were incubated with HUVECs. Confocal microscopy revealed punctate perinuclear signals co‐localized with the actin cytoskeleton, confirming migrasome internalization (Figure S4). We next assessed the angiogenic effects of microglia‐derived migrasomes in vivo and in vitro. Equal amounts of isolated migrasomes were administered in each treatment group. Migrasomes isolated from BV2 microglia exposed to HG, hypoxia, or their combination were intravitreally injected into OIR mice at postnatal day 12 (P12) (Figure 5A). IB4 staining of retinal flat mounts showed increased neovascular tuft area following migrasome administration, with the most pronounced effect observed in mice receiving migrasomes derived from combined HG and hypoxic stress (Figure 5B,C). In parallel, HUVECs were treated with migrasomes isolated from HMC3 cells under the corresponding conditions. CCK‐8 and EdU assays showed that migrasomes derived from combined stress boosted endothelial proliferation and cellular activity (Figure 5D–G). HUVEC migration was significantly increased only in the combined‐stress migrasome group (Figure 5H,I). Tube formation assays further showed that migrasomes from all treatment groups promoted vascular network formation, with the greatest increases in junction number and total segment length observed in the combined‐stress group (Figure 5J–L). Together, these findings demonstrate that microglia‐derived migrasomes are sufficient to enhance endothelial angiogenic responses and pathological RNV, with migrasomes generated under combined HG and hypoxic stress exhibiting the strongest effects.

### 2.6 Microglial Migrasomes Activate Endothelial HIF‐1α/VEGF Signaling

**FIGURE 6:**
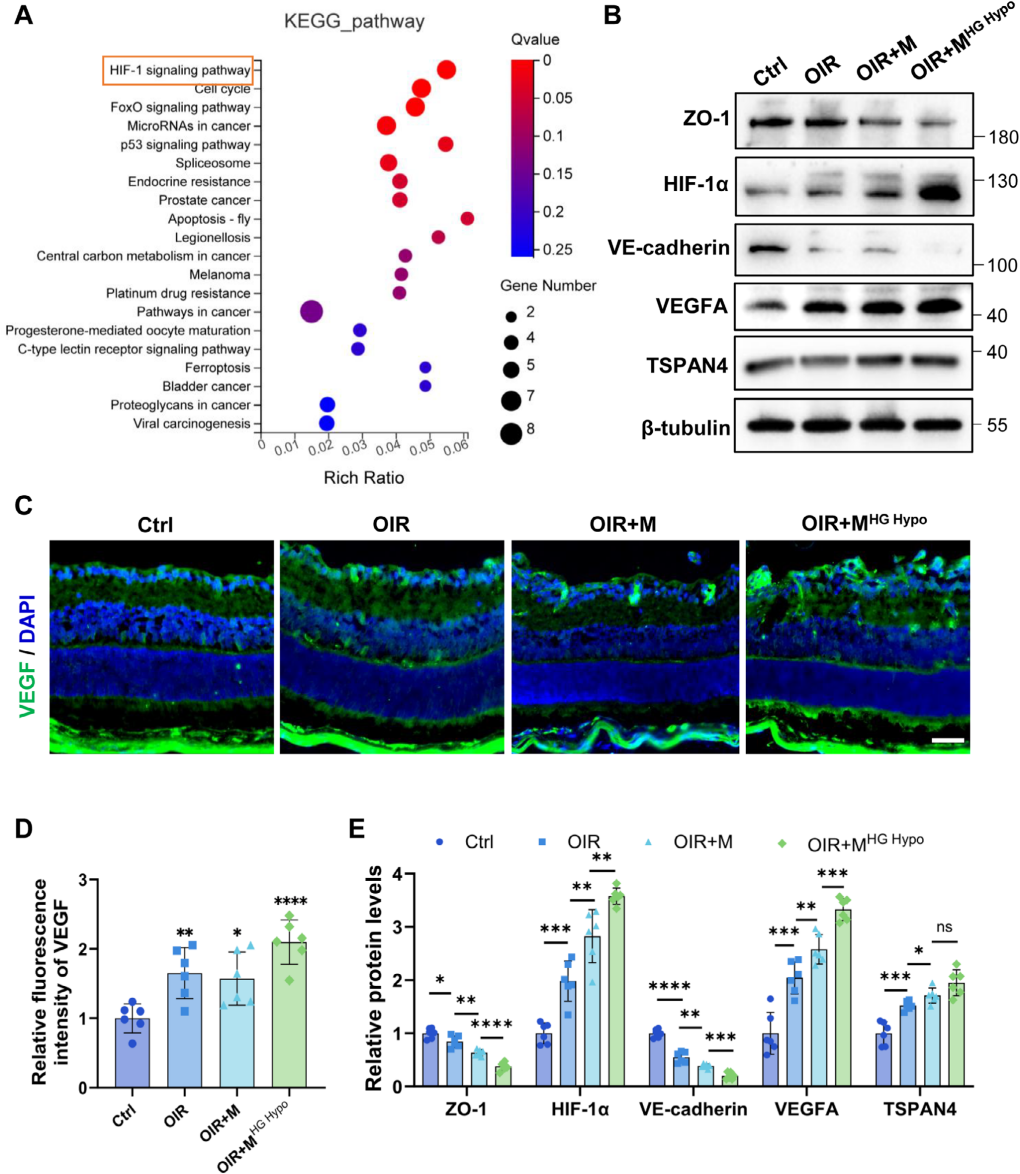
Migrasomes from stressed microglia activate the HIF‐1α/VEGF pathway in OIR. (A) KEGG pathway enrichment analysis of differentially expressed genes in HUVECs treated with migrasomes derived from control or combined HG‐ and hypoxia‐treated microglia, identifying HIF‐1 signaling as one of the most significantly enriched pathways. (B) Western blot analysis of retinal lysates from control mice, OIR mice, OIR mice injected with unstimulated migrasomes (OIR+M), and OIR mice injected with stressed migrasomes (OIR+M^HG Hypo^). (C) Representative immunofluorescence staining of VEGF in retinal sections, showing enhanced VEGF expression within neovascular regions, particularly in the OIR+M^HG Hypo^ group. Scale bar = 50 µm. (D) Quantification of VEGF fluorescence intensity shown in (C). (E) Quantification of protein levels in (B) (n = 6). Data are presented as mean ± SD. Comparisons among multiple groups were performed using one‐way or two‐way ANOVA followed by Tukey's post–hoc test, as appropriate. ^*^ *p* < 0.05; ^**^ *p* < 0.01; ^***^ *p* < 0.001; ^****^ *p* < 0.0001.

**FIGURE 7:**
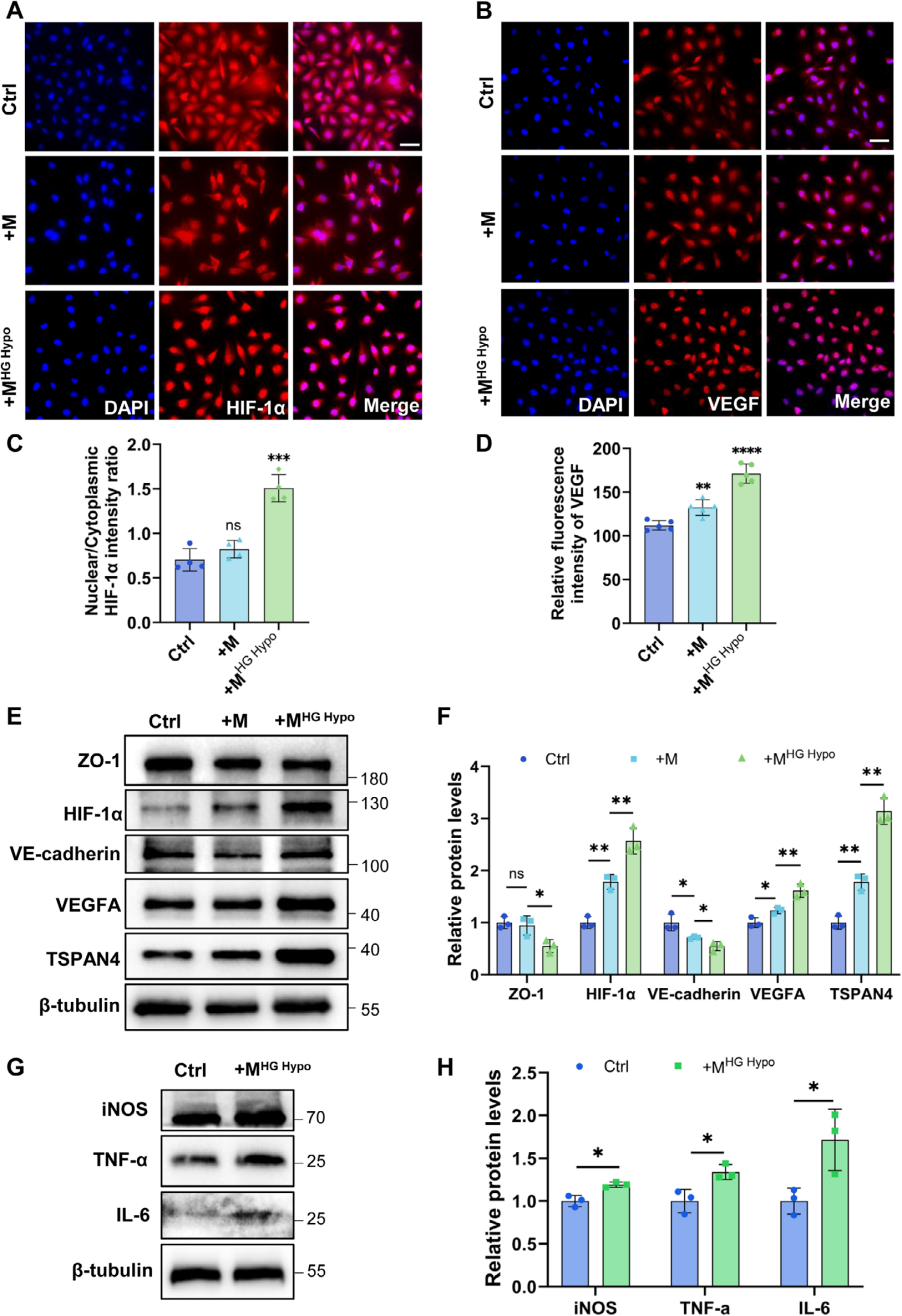
Migrasomes from stressed microglia activate endothelial HIF‐1α signaling and reinforce microglial inflammation. (A) Immunofluorescence staining of HIF‐1α (red) in HUVECs treated with migrasomes derived from unstimulated microglia (+M) or combined HG‐ and hypoxia‐treated microglia (+M^HG Hypo^). Nuclear HIF‐1α accumulation was enhanced in the +M^HG Hypo^ group. Scale bar = 50 µm. (B) Immunofluorescence staining of VEGF (red) in HUVECs under the indicated conditions, showing increased VEGF expression in the +M^HG Hypo^ group. Scale bar = 50 µm. (C) Quantification of HIF‐1α nuclear‐to‐cytoplasmic ratio in (A). (D) Quantification of VEGF fluorescence intensity in (B). (E) Western blot analysis of HIF‐1α, VEGFA, ZO‐1, and VE‐cadherin in HUVECs treated with the indicated migrasome preparations. (F) Quantification of the indicated protein levels in (E). (G) Western blot analysis of microglia treated with migrasomes derived from microglia exposed to combined HG and hypoxia, showing elevated iNOS, TNF‐α, and IL‐6 expression. (H) Quantification of the indicated protein levels in (G). Data are presented as mean ± SD. Two‐group comparisons were analyzed by unpaired two‐tailed Student's *t*‐test; multi‐group comparisons were analyzed by one‐way or two‐way ANOVA followed by Tukey's post‐hoc test, as appropriate. ^*^ *p* < 0.05; ^**^ *p* < 0.01; ^***^ *p* < 0.001; ^****^ *p* < 0.0001.

To identify the endothelial signaling pathways mediating the angiogenic effects of microglial migrasomes, we performed transcriptomic profiling of HUVECs treated with migrasomes derived from combined HG‐ and hypoxia‐treated microglia. Pathway enrichment analysis identified HIF‐1α signaling as one of the most significantly enriched pathways (Figure 6A). This pathway activation was validated in vivo using the OIR model, where intravitreal administration of migrasomes derived from stressed microglia (OIR+M^HG Hypo^) markedly increased retinal HIF‐1α and VEGFA protein levels while downregulating key vascular barrier components, including VE‐cadherin and ZO‐1 (Figure 6B,E). Immunofluorescence staining further confirmed a robust elevation of VEGF immunoreactivity within pathological neovascular tufts following migrasome administration (Figure 6C,D). Consistent with these in vivo observations, treatment with stressed microglial migrasomes induced prominent nuclear translocation of HIF‐1α in HUVECs (Figure 7A,C), a functional hallmark of its transcriptional activation, which was accompanied by enhanced VEGFA expression (Figure 7B,D). Western blot analysis corroborated the activation of the HIF‐1α/VEGF axis alongside the concomitant loss of endothelial barrier markers (Figure 7E,F). Beyond their direct pro‐angiogenic actions on endothelial cells, these migrasomes also triggered a pro‐inflammatory shift in recipient microglia, as evidenced by elevated expression of iNOS, TNF‐α, and IL‐6 (Figure 7G,H). This suggests that migrasomes intrinsically reinforce microglial activation, establishing a pathogenic feed‐forward loop that amplifies retinal injury. Collectively, these findings demonstrate that migrasomes derived from metabolically and hypoxically stressed microglia activate endothelial HIF‐1α/VEGF signaling, compromise vascular integrity, and amplify microglial inflammatory responses, thereby establishing a mechanistic bridge between microglial migrasome biogenesis and pathological RNV.

## 3 Discussion

Pathological retinal neovascularization is a vision‐threatening hallmark of PDR and reflects dysfunction of the retinal neurovascular–immune unit rather than an isolated vascular abnormality. In this study, we identified a continuous TSPAN4‐associated microglial state in human PDR fibrovascular membranes and murine OIR retinas. TSPAN4‐detected microglia exhibited higher scores for a broader TSPAN4‐associated transcriptional signature and were distributed across the microglial landscape. This signature partially overlapped with established activated and disease‐associated microglial programs but did not correspond to a single canonical subtype. At the tissue level, TSPAN4‐expressing microglia accumulated within and around pathological neovascular lesions. Together with the reduction in neovascularization observed in Tspan4^−/−^ mice, these findings associate TSPAN4‐dependent microglial activity with pathological vascular remodeling.

Microglia are conventionally thought to regulate endothelial behavior primarily through soluble mediators such as VEGF, TNF‐α, and IL‐1β [15, 39]. However, this diffusive signaling model inadequately explains the focal and directional nature of pathological neovascular tufts. Our findings reveal that microglia communicate with endothelial cells via migrasomes—a novel class of organelles mediating intercellular communication. Migrasome formation is coupled to cell migration, enabling these structures to serve as vehicles for signal transfer between cells [40, 41, 42]. As micron‐scale signaling platforms [43, 44, 45], migrasomes offer spatial precision and signaling efficiency [46, 47]. Although migrasomes have been investigated in tumor progression, tissue injury, and neurological disease [48, 49, 50, 51, 52], their contribution to retinal pathology remains poorly understood. This study provides evidence that migrasomes deposited along microglial migration trajectories within neovascular retinas act as signaling stations, delivering pro‐angiogenic cargo directly to adjacent endothelial cells. This conceptual shift from diffuse secretion to directed delivery offers an explanation for the spatial coincidence of stressed microglia with neovascular tufts and reveals a previously unrecognized mode of intercellular communication in the retina.

TSPAN4 is a fundamental regulator of migrasome biogenesis and downstream signaling. In cultured microglia, TSPAN4 overexpression significantly enhanced endothelial proliferation, migration, and network formation, whereas TSPAN4 knockdown exerted the opposite effects. In parallel, global Tspan4 deficiency markedly attenuated pathological neovascularization in both OIR and laser‐induced CNV models. Because TSPAN4 is expressed across multiple cell types and the knockout model utilized here was global, these in vivo findings do not definitively establish a strictly cell‐autonomous effect restricted to microglia. Nevertheless, the convergence of our genetic, co‐culture, and functional migrasome‐transfer experiments strongly supports the critical involvement of the microglial TSPAN4–migrasome axis in pathological angiogenesis. Crucially, targeting TSPAN4‐mediated migrasome formation represents an upstream point of intervention that attenuates immune–vascular crosstalk at its source, setting it apart from direct VEGF neutralization [53, 54]. While this axis offers a novel therapeutic perspective—potentially complementing existing anti‐VEGF regimens—its cellular selectivity, safety profile, and translational feasibility warrant comprehensive further evaluation.

Proteomic analysis further showed that migrasomes generated under combined high‐glucose and hypoxic stress had an altered cargo profile enriched in angiogenesis‐ and matrix‐remodeling‐associated proteins, including FN1, THBS1, ECM1, IGF2, and ANXA2/5. Functionally, administration of stress‐derived migrasomes increased neovascular tuft formation in OIR retinas and enhanced endothelial proliferation, migration, and tube formation in vitro. Transcriptomic profiling of migrasome‐treated endothelial cells identified enrichment of the HIF‐1 signaling pathway (Figure 6A). Consistently, migrasome exposure increased endothelial HIF‐1α nuclear accumulation and VEGFA expression while reducing the junction‐associated proteins ZO‐1 and VE‐cadherin. These results indicate that stressed microglial migrasomes activate endothelial HIF‐1α/VEGF signaling in association with angiogenic activation and barrier destabilization. However, pathway inhibition or rescue experiments will be required to determine whether HIF‐1α activation is necessary for the observed angiogenic effects.

Migrasome signaling may also extend beyond direct effects on endothelial cells. Exposure of recipient microglia to stress‐derived migrasomes increased the expression of iNOS, TNF‐α, and IL‐6, suggesting reinforcement of an inflammatory microglial response (Figure 7G,H). We therefore propose a potential feed‐forward mechanism in which pathological stress enhances TSPAN4‐dependent migrasome formation, these migrasomes activate endothelial angiogenic signaling, and their effects on surrounding microglia may further amplify local inflammation. Although this model provides a framework linking immune activation with vascular remodeling, its temporal sequence and relevance in vivo remain to be established.

Several limitations of this study should be acknowledged. First, the global Tspan4 knockout model cannot fully rule out the potential contribution of TSPAN4 from other retinal cell types; future studies using microglia‐specific conditional knockouts (Cx3cr1‐Cre) will be critical to definitively establish cell‐autonomous mechanisms. Second, while uptake and intravitreal transfer experiments demonstrate the functional potency of isolated migrasomes, direct in vivo visualization and real‐time tracking of endogenous microglial migrasomes within neovascular lesions remain technically challenging and warrant further development. Third, although proteomic profiling revealed stress‐induced enrichment of pro‐angiogenic cargo, side‐by‐side comparison with matched donor‐cell proteomes will be necessary to determine whether these molecules are selectively sorted into migrasomes. Fourth, further loss‐ or gain‐of‐function studies targeting HIF‐1α/VEGF signaling will be required to firmly establish its absolute causality downstream of migrasome uptake. Finally, the potential involvement of migrasomes derived from other retinal constituents—such as Müller cells, astrocytes, and pericytes—warrants systematic exploration. Despite these current boundaries, given their endogenous biocompatibility and target specificity, microglial migrasomes may ultimately inspire new paradigms for targeted therapeutic delivery [55, 56] in ocular neovascular diseases.

In conclusion, our study identifies a continuous TSPAN4‐associated microglial state linked to pathological retinal neovascularization and implicates TSPAN4‐dependent migrasome formation as a mechanism of microglia–endothelial communication. Stress‐derived microglial migrasomes enhance endothelial angiogenic responses, activate HIF‐1α/VEGF signaling, and may reinforce local microglial inflammation. These findings expand the current framework of immune–vascular crosstalk in retinal disease and support further investigation of the TSPAN4–migrasome axis as a potential therapeutic target.

## 4 Materials and Methods

### 4.1 Clinical Sample Collection

To elucidate the potential impact of microglia‐derived migrasomes on DR, fibrovascular membranes were collected from patients diagnosed with PDR. All specimens were harvested from PDR patients undergoing vitrectomy at The First Affiliated Hospital of Nanjing Medical University between June and December 2024 (n = 6). Patients with a history of prior anti‐VEGF therapy were excluded. Surgically excised tissues were immediately fixed, dehydrated, and embedded, followed by preparation of frozen sections for immunofluorescence analysis. Human study protocols aligned strictly with the ethical standards of the Declaration of Helsinki and received formal clearance from the Institutional Ethics Committee of Nanjing Medical University (Approval No. 2022‐SR‐224), with written informed consent secured from all enrolled subjects.

### 4.2 Animal Models and Experimental Treatments

C57BL/6J mice sourced from Beijing Vital River Laboratory Animal Technology were housed in the Nanjing Medical University Animal Core Facility, maintained under controlled environmental settings (22°C ± 1°C, 12 h light/dark illumination cycle) with ad libitum food and water supply. All animal procedures were approved by the Animal Ethics Committee of Nanjing Medical University (IACUC‐2404012).

Oxygen‐induced retinopathy (OIR) model: Neonatal C57BL/6J pups and their nursing mothers were exposed to 75% oxygen from postnatal day 7 (P7) to P12, followed by a return to room air at P12. At P12, OIR mice received intravitreal injections of 1.5 µL PBS or migrasomes after anesthesia. All intravitreal injections used a 5 µL Hamilton syringe with a 33‐gauge needle. Normoxic littermates kept continuously in room air served as age‐matched controls. Retinal tissues were collected at P17 for downstream evaluation.

Laser‐induced choroidal neovascularization (CNV) model: 6–8‐week‐old C57BL/6J mice were anesthetized via intraperitoneal injection of 1.25% tribromoethanol (0.25 mL/10 g). Photocoagulation was executed using a 532 nm laser system (Iris Radiation Systems, USA; 100 mW power, 100 ms duration, 75 µm spot diameter) to generate four burns circumscribing the optic nerve head at approximately two‐disc diameters away. To prevent ocular infection, ofloxacin eye ointment was applied topically post‐procedure. Choroidal tissues were harvested 7 days later for further analyses.

### 4.3 Cell Culture and Treatment

Human umbilical vein endothelial cells (HUVECs), the murine microglial cell line BV2, and human microglial clone 3 (HMC3) cells were purchased from ATCC. HUVECs were cultured in Endothelial Cell Medium (ECM, ScienCell), BV2 cells in RPMI 1640 (Gibco) and HMC3 cells in Minimum Essential Medium (MEM, Gibco). All basal media were fortified with 10% fetal bovine serum (FBS) and 1% penicillin–streptomycin (P/S). Cultures were maintained in a humidified atmosphere containing 5% CO_2_ at 37°C.

To recapitulate the microenvironment of DR in vitro, cells were exposed to high glucose (HG, 30 mmol/L) compared with normal glucose controls (NG, 5.5 mmol/L), or subjected to hypoxic conditions (1% O_2_) for 48 h utilizing a Forma Steri‐Cycle i160 tri‐gas incubator (Thermo Fisher Scientific).

### 4.4 Live‐Cell Time‐Lapse Imaging

For dynamic tracking of migrasome formation, cells were cast onto the central glass microwell of confocal dishes at a density of 2 × 10^4^ cells per dish. After adherence, cells received designated treatments and were membrane‐labeled with wheat germ agglutinin (WGA; Invitrogen, W11262). Sequential time‐lapse imaging was executed on a high‐sensitivity structured illumination microscope (HIS‐SIM, China) with frame captures set at 5‐min intervals. All live‐cell imaging was conducted under physiological temperature and CO_2_ conditions.

### 4.5 Lentiviral Transduction and Gene Silencing/Overexpression

TSPAN4 expression was stably knocked down or overexpressed in HMC3 cells via lentiviral delivery systems synthesized by GenePharma (Shanghai, China). Constructs included TSPAN4‐targeting shRNA (Lv‐shTSPAN4‐GFP; control: Lv‐shNC) and full‐length TSPAN4 cDNA (Lv‐TSPAN4‐OE‐GFP; control: Lv‐NC‐OE).

Among three synthesized shRNAs, the sequence achieving maximal interference efficiency was chosen for subsequent assays. At 48 h post‐infection, green fluorescent protein (GFP) expression was verified under a fluorescence microscope, and transduced cells were collected for validation and functional assays. Target sequences are listed in Table S3.

### 4.6 Endothelial–Microglial Co‐Culture Assay

Endothelial‐microglial crosstalk was evaluated using a Transwell co‐culture system. HUVECs (1 × 10^5^ cells/well) were seeded in the lower chamber, while pretreated HMC3 cells (5 × 10^4^ cells/insert) were placed into the upper porous inserts. Equal serum concentrations were maintained in both compartments to preclude serum‐gradient confounders. After 24 h of co‐cultivation, HUVECs were harvested for phenotypic and molecular analyses.

### 4.7 Isolation and Characterization of Migrasomes and Extracellular Vesicles

Microglial cells (HMC3 for in vitro assays; BV2 for in vivo murine studies to avoid interspecies immune interference) at ∼50% confluence were harvested together with their culture medium. Sequential centrifugation at 1000 × g for 10 min and 4000 × g for 20 min at 4°C removed intact cells and cellular debris. The supernatant was spun at 20 000 × g for 30 min to pellet crude migrasomes, which were purified using a density gradient centrifugation kit (Sigma–Aldrich) at 150 000 × g for 4 h, followed by a final washing step in PBS at 20 000 × g for 30 min.

For extracellular vesicle (EV) extraction, conditioned media underwent centrifugation (300 × g for 10 min, 2000 × g for 10 min, and 8000 × g for 30 min), followed by filtration through a 0.22‐µm membrane. EVs were collected by ultracentrifugation at 100 000 × g for 70 min, washed in PBS, and repelleted at 100 000 × g for an additional 70 min at 4°C.

For transmission electron microscopy (TEM), purified migrasomes were fixed, adsorbed onto carbon‐coated copper grids, negatively stained, and imaged on a Hitachi HT7700 microscope.

### 4.8 Western Blotting

Total proteins were extracted with RIPA buffer containing protease inhibitors, quantified via BCA assay (Thermo Fisher Scientific, USA), separated by SDS–PAGE, and transferred onto 0.22 µm PVDF membranes (Millipore, USA). Membranes were blocked with 5% non‐fat milk, incubated with primary antibodies overnight at 4°C, washed with TBST, and probed with HRP‐conjugated secondary antibodies for 1 h at room temperature prior to ECL imaging (Tanon 5200, China). Detailed information regarding all primary and secondary antibodies, including manufacturers, catalog numbers, host species, and dilution ratios, is provided in Table S4.

### 4.9 Proliferation Assays

HUVECs (5 × 10^3^ cells/well) were seeded into 96‐well plates. Cell viability was monitored at 24, 48, and 72 h using a CCK‐8 assay kit (Biosharp; 2 h incubation at 37°C; A_450nm_ read on a Thermo Fisher microplate reader). DNA synthesis capability was evaluated using the BeyoClick EdU‐594 Cell Proliferation Kit (Beyotime, Shanghai, China). HUVECs were incubated with 10 µM EdU working solution for 2 h at 37°C, followed by 4% PFA fixation and Triton X‐100 permeabilization. After washing, cells were incubated with the Click reaction cocktail for 30 min in the dark and counterstained with DAPI for 5 min to visualize total nuclei.

### 4.10 Transwell Migration

HUVEC migration was evaluated using Transwell inserts (8.0‐µm pore size; Corning, USA). Cells (4 × 10^4^ cells/well) in serum‐free medium were seeded into the upper chamber, with medium containing 20% FBS added to the lower chambers as a chemoattractant. After 24 h, transmigrated cells on the lower membrane surface were fixed with 4% PFA, stained with 0.1% crystal violet, and quantified under an inverted microscope (Olympus, Japan).

### 4.11 Matrigel Tube Formation

Pre‐chilled 96‐well plates were coated with Growth Factor Reduced Matrigel (50 µL/well; Corning, USA) and polymerized at 37°C for 30 min. HUVECs (1.5 × 10^4^ cells/well) were seeded on Matrigel. Tubular network structures were captured at 6–8 h using an inverted microscope (Olympus, Japan). Total tube length and junction numbers were quantified using ImageJ software with the Angiogenesis Analyzer plugin.

### 4.12 Immunofluorescence Staining

Cryosections (10 µm) or fixed cells were permeabilized with 0.5% Triton X‐100 for 15 min and blocked with 5% BSA for 1 h. Samples were then incubated with primary antibodies overnight at 4°C. After PBS washes, specimens were incubated with appropriate fluorophore‐conjugated secondary antibodies for 1 h at room temperature in the dark, followed by DAPI counterstaining for 5 min to visualize nuclei. Detailed antibody sources and dilution ratios are provided in Table S4. Images were captured using a fluorescence microscope (Leica THUNDER DMi8, Germany).

### 4.13 RNA Sequencing and Bioinformatic Analysis

HUVECs were divided into control (Ctrl) and migrasome‐treated groups (+M^HG Hypo^), each with three biological replicates (n = 3). Total RNA was extracted using TRIzol reagent (Invitrogen, USA). Poly(A) + mRNA enrichment, cDNA library construction, and paired‐end 150‐bp sequencing (PE150) were performed on the DNBSEQ platform (BGI, Shenzhen, China) following standard eukaryotic transcriptome protocols. Each library yielded approximately 6.62 Gb of clean data, with Q30 quality scores consistently exceeding 96.86%. After quality control and read alignment, differentially expressed genes (DEGs) were identified with a threshold of *Q* value ≤ 0.05, followed by Gene Ontology (GO) and Kyoto Encyclopedia of Genes and Genomes (KEGG) enrichment analyses for functional annotation.

### 4.14 Single‐Cell RNA Sequencing Data Processing and Quality Control

Publicly available scRNA‐seq datasets for DR and the OIR model were obtained from the GEO database under accession numbers GSE165784 and GSE152928, respectively. Gene–cell count matrices were processed in R using Seurat (v5.5.0) and SeuratObject (v5.0.2). Genes detected in fewer than three cells were excluded (min.cells = 3). For human FVM samples, cells with 500 – 5000 detected genes (nFeature_RNA) and < 20% mitochondrial transcripts (percent.mt) were retained. For mouse OIR samples, cells with > 200 detected genes and < 15% mitochondrial transcripts were retained. No fixed UMI count (nCount_RNA) threshold was applied. Mitochondrial transcript proportions were calculated using genes prefixed with “MT‐” in the human dataset and “mt‐” in the mouse dataset. Ambient RNA contamination in the OIR dataset was assessed using decontX implemented in celda (v1.22.1). Doublets were identified and removed using DoubletFinder (v2.0.4) before downstream analysis. Quality‐control metrics before and after filtering, sample‐wise DoubletFinder results, sample representation, cluster composition, and sample‐level QC distributions for the FVM dataset are shown in Figure S5. Following quality control, the datasets were normalized, variable features were identified, and principal component analysis was performed using Seurat. Human FVM samples were merged, and inter‐sample batch effects were corrected using Harmony (v1.2.3) on the principal‐component embeddings. The resulting Harmony embeddings were used for nearest‐neighbor graph construction, clustering, and UMAP visualization. Major cell lineages and subclusters were annotated independently of TSPAN4 expression based on established lineage‐specific marker genes. Subsequent analyses of the TSPAN4‐associated state were restricted to cells annotated as microglia. Differential expression analysis and module scoring were performed using Seurat. Additional packages used for data manipulation, sparse‐matrix operations, statistical summaries, and visualization included Matrix (v1.7.1), dplyr (v1.1.4), tidyr (v1.3.1), tibble (v3.2.1), ggplot2 (v4.0.3), patchwork (v1.3.0), and scales (v1.4.0) (Table S5).

### 4.15 Statistical Analysis

All experiments were repeated at least three times independently. Data are presented as mean ± standard deviation (SD). Statistical analyses were performed using GraphPad Prism 10.1.2 (GraphPad Software, USA). Comparisons between two groups were conducted using Student's *t*‐test, while comparisons among three or more groups were performed using one‐way or two‐way analysis of variance (ANOVA) as appropriate. A *P* value < 0.05 was considered statistically significant.

## Author Contributions

J.X. wrote the manuscript. J.X., J.W. and Q.G. performed the animal experiments. J.X. and J.W. contributed to the isolation of migrasomes. P.G. analyzed the single‐cell RNA sequencing data. J.W. and Y.F. collected the clinical samples. H.L. and H.X. contributed to the analysis of the data. P.X. and Z.H. contributed to the study conception, design, funding and manuscript revision. All authors commented on previous versions of the manuscript. All authors read and approved the final manuscript.

## Funding

This work was supported by the National Natural Science Foundation of China (82371076 to P.X., 82471095 to Z.H.), Interdisciplinary Convergence of Medical Engineering Project of the First Affiliated Hospital of Nanjing Medical University (QG202405), and the Social Development Program of Jiangsu Province (BE2021744 to P.X.).

## Ethics Statement

All animal studies were conducted in accordance with the ARVO Statement for the Use of Animals in Ophthalmic and Vision Research and were approved by the Institutional Animal Care and Use Committee at Nanjing Medical University (IACUC‐2404012). All human procedures were conducted in accordance with the Declaration of Helsinki and were approved by the Ethics Committee of Nanjing Medical University (Approval No. 2022‐SR‐224). Written informed consent was obtained from all participants.

## Conflicts of Interest

The authors declare no conflicts of interest.

## Supporting information




**Supporting File 1**: advs77711‐sup‐0001‐SuppMat.docx.

**Supporting File 2**: advs77711‐sup‐0002‐SuppMat.xlsx.

**Supporting File 3**: advs77711‐sup‐0003‐S1.xlsx.

**Supporting File 4**: advs77711‐sup‐0004‐S2.xlsx.

**Supporting File 5**: advs77711‐sup‐0005‐S3.xlsx.

**Supporting File 6**: advs77711‐sup‐0006‐S4.xlsx.

**Supporting File 7**: advs77711‐sup‐0007‐scRNAseq.xlsx.

## Data Availability

The data that support the findings of this study are available in the supplementary material of this article.
