# Supplementary material for: Microglial TSPAN4‐Dependent Migrasomes Promote Pathological Retinal Neovascularization via Immune‐Vascular Crosstalk
Source: Adv Sci (Weinh). 2026 Sep 13:e77711. Online ahead of print. doi: 10.1002/advs.77711 (PMC13572864; doi:10.1002/advs.77711)
Supplement: Supplementary file 1 [file ADVS-9999-e77711-s007.docx]

**Microglial TSPAN4-Dependent Migrasomes Promote Pathological Retinal Neovascularization via Immune-Vascular Crosstalk**

Jingyi Xu^1^, Jingfan Wang^1^, Pengfei Ge^1^, Qinyuan Gu^1^, Hongying Li^1^, Yuanyuan Fan^1^, Haiyue Xie^1^, Yangyang Lu^1^, Yifan Lin^1^, Xinjing Wu^2^, Chengkun Wang^3^, Ping Xie^1, *^ and Zizhong Hu^1, *^

^1^*Department of Ophthalmology, The First Affiliated Hospital of Nanjing Medical University, Nanjing 210029, China.*

*^2^Department of Ophthalmology, Children's Hospital of Nanjing Medical University, Nanjing 210093, China*

*^3^Department of Physiology, School of Basic Medical Sciences, Nanjing Medical University, Nanjing, 211166, China.*

Jingyi Xu and Jingfan Wang contributed equally to this work.

**Corresponding Author:**

**Zizhong Hu**

Department of Ophthalmology, The First Affiliated Hospital of Nanjing Medical University, Nanjing, 300 Guangzhou Road, 210029, China, huzizhong@njmu.edu.cn.

**Ping Xie**

Department of Ophthalmology, The First Affiliated Hospital of Nanjing Medical University, Nanjing, 300 Guangzhou Road, Nanjing, 210029, China, xieping9@126.com.

**Supplementary Figure 1 Definition and transcriptional characterization of the TSPAN4-associated microglial state in human PDR fibrovascular membranes**

(A) Relative proportions of the major annotated cell types in each of the five human FVM samples. (B) Proportions of TSPAN4-detected microglia in the five FVM samples. (C) Violin plots comparing TSPAN4-associated signature scores between TSPAN4-detected and TSPAN4-undetected microglia within each sample. (D) Heatmap showing the log₂ fold changes of the 30 TSPAN4-associated signature genes between TSPAN4-detected and TSPAN4-undetected microglia across the five samples. (E) Heatmap showing the standardized scores of established proliferative, LPS-associated inflammatory, interferon, homeostatic, and APOE-associated disease-associated microglial programs across quartiles of the TSPAN4-associated signature score. Q1 and Q4 represent microglia with the lowest and highest TSPAN4-associated signature scores, respectively.

**Supplementary Figure 2 Validation of *Tspan4* knockout efficiency**

(A) Immunofluorescence staining of retinal sections from OIR^KO^ and OIR^WT^ mice, confirming effective Tspan4 deletion. Scale bar = 50 μm. (B) Western blot analysis of TSPAN4 protein expression in retinal lysates from WT and KO mice. (C) Quantification of TSPAN4 protein in (B). Data are presented as mean ± SD. Statistical significance was determined using unpaired two-tailed Student's t-test. **p* < 0.05; ***p* < 0.01; ****p* < 0.001; *****p* < 0.0001.

**Supplementary Figure 3 Comparative Proteomic Profiling of Microglia-Derived Migrasomes Under Pathological Stress**

(A) Volcano plot illustrating differentially expressed proteins (DEPs) in migrasomes isolated from microglia exposed to hyperglycemic and hypoxic (HG Hypo) conditions compared to control (Ctrl) conditions. Red dots represent significantly upregulated proteins, while blue dots represent significantly downregulated proteins (*P* < 0.05). (B) Hierarchical clustering heatmap showcasing the enriched expression levels of selected pathological regulators associated with extracellular matrix remodeling, growth factor signaling, and immune activation (including Fn1, Thbs1, Ecm1, Igf2, Igfbp2–4, Anxa2/5, C1qc, and C1rb) across three biological replicates per group. Color scale indicates relative expression levels (Z-score transformed).

**Supplementary Figure 4 Uptake of microglia-derived migrasomes by endothelial cells in vitro**

PKH26-labeled migrasomes (red) isolated from stressed microglia were co-incubated with HUVECs. After incubation, cells were fixed and stained for F-actin (green) and nuclei (DAPI, blue). Confocal microscopy images show punctate PKH26 signals in the perinuclear region, which co-localize with the actin cytoskeleton, indicating successful uptake of migrasomes by recipient endothelial cells. Scale bar = 10 μm.

**Supplementary Figure 5 Quality-control assessment and sample composition of human FVM scRNA-seq datasets**

(A) Distributions of the number of detected genes (nFeature_RNA), UMI counts (nCount_RNA), and mitochondrial transcript percentage (percent.mt) before and after quality-control filtering in each of the five FVM samples. (B) DoubletFinder classification results showing predicted singlets and doublets in each FVM sample. (C) Harmony-integrated UMAP of the retained FVM singlets, colored by sample of origin. (D) Sample composition of the annotated FVM cell clusters, showing the relative contribution of each of the five samples to individual clusters. (E) Comparison of quality-control metrics among the retained singlets across the five FVM samples.
